# Supplementary material for: Position-dependent plasticity of distinct progenitor types in the primitive streak
Source: eLife. 2016 Jan 18;5:e10042. doi: 10.7554/eLife.10042 (PMC4798969; doi:10.7554/eLife.10042)
Supplement: Figure 6—source data 1. — Additional information and analysis to show the majority of paraxial mesoderm in CLE grafts is derived from NMPs rather than contaminating PSM progenitors that were co-grafted. DOI: http://dx.doi.org/10.7554/eLife.10042.011 [file elife-10042-fig6-data1.pdf]

Figure 6-figure supplement 5: Presomitic Mesoderm Contamination in CLE Grafts.

In our previous investigation of CLE fate [1] the underlying presomitic mesoderm from CLE grafts was not removed and almost all grafted embryos contained a short (3-8 somite) unilateral stretch of graft-derived paraxial mesoderm rostral to a stretch of unilateral neural and bilateral mesoderm contribution. Importantly, the unilateral mesoderm label was located more rostrally than the rostral limit of labelling of grafts that were derived from ingressing mesoderm (i.e. homotopic primitive streak grafts). We therefore concluded that the unilateral stretch of labelled mesoderm was likely to have arisen from pre-existing PSM grafted together with the CLE ([1], Figure 5). Therefore, we attempted to minimise this contamination in the current work by removing mesoderm underlying the CLE before grafting. Figure 1 below shows the workflow for removal of mesoderm.

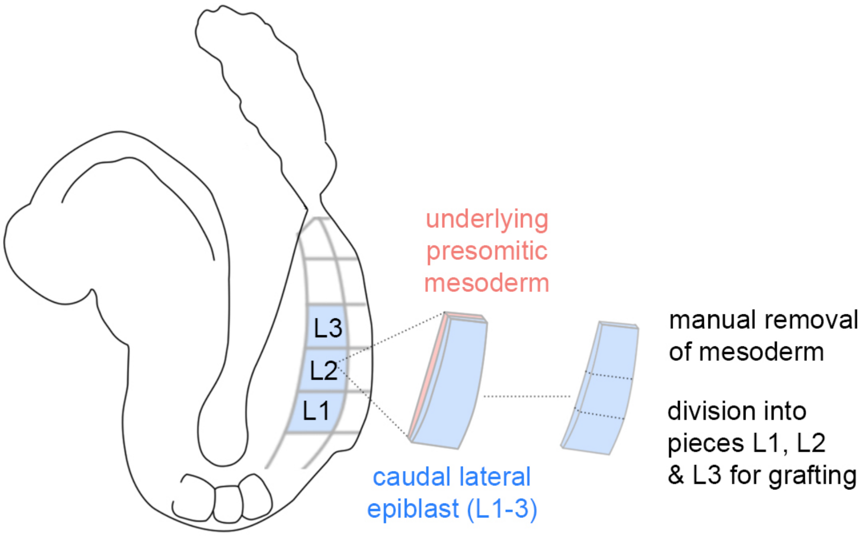

Figure 1. Workflow for removal of presomitic mesoderm in CLE grafts. CLE grafts correspond to about 100-150 cells for each region (L1, L2 or L3). Cells were dissected and grafted as described [1] with the exception that the underlying mesoderm was manually dissected away from the ectoderm using hand-pulled solid glass needles before grafting.

We chose to manually remove mesoderm rather than enzymatically separate germ layers to ensure a faster workflow and therefore better tissue and embryo viability. This inevitably left a small amount of pre-existing mesoderm. **It is therefore important to rule out the possibility that a significant proportion of the mesoderm contribution that we observe is derived from pre-existing mesoderm, rather than neuromesodermal progenitor (CLE)-derived mesoderm.** The considerations below allow us to rule out this possibility.

Firstly, underlying mesoderm is estimated to be a minor contaminant since the unilateral stretch of mesoderm in the current grafting series is much less prominent than in *Cambray and Wilson, 2007* (compare Figure 5F in [1] and Figure 6Aa, Am and Aq in this work). A clear separate anterior stretch of unilateral mesoderm was observed in only 5/29 homotopic L1-3 grafts. This suggests that removal of underlying mesoderm was effective.

Secondly, the mesoderm carried along with the graft is, by virtue of its position immediately underlying the CLE, paraxial (presomitic) mesoderm. This is known to contribute only to short stretches of the axis, i.e. less than 6 somites, as measured in three ways:

- WGA-gold labelling of cells in different fractions of the presomitic mesoderm has shown that the anterior and mid aspect of the E8.5 PSM give rise to a distinct and limited contribution of 3 to 6 somites, respectively [2].

- *Nicolas et al.* [3] describe 'short', presumably PSM-derived clones that contribute to 1-3 somites and are always unilateral; those that contribute to 4-6 somites that are likely to be their immediate progenitors, are mostly bilateral, and long clones, presumably derived from NMPs, are almost always bilateral. This strongly implies that PSM cells do not transgress more than two somite boundaries after exiting the midline.

- A comparison of the development of E8.5, E9.5 and E10.5 PSM explants with or without the caudal progenitor region has shown that explants lacking the caudal region (tail bud) form significantly fewer somites than those including the caudal region. Moreover, in E8.5-E10.5 PSM explants that were devoid of the caudal progenitor region, only ~5-6 somites were formed [4].

In homotopic grafts of CLE (L1-3) in this study, we observed contribution to many more somites than this. The average rostral limit of contribution in these embryos is 16.7±4.9; and since most (25/29) CLE grafts developed at least the PSM corresponding to somite 28 (level with the caudal edge of the hindlimb bud), this corresponds to a labelled stretch of at least 11 somites. Therefore, the bulk of the mesoderm formed in CLE grafts cannot be derived from pre-existing presomitic mesoderm carried along with the graft.

Finally, while some stretches of unilateral mesoderm contribution were observed in all 29 homotopic CLE grafts, unilateral contribution could come from either the CLE or pre-existing mesoderm. However, any bilateral mesoderm contribution indicates that the progenitors of those cells had encountered the midline primitive streak at some point in their history [3, 5]. This is the case for 18/22 grafts containing putative NMPs (5/6 L1AT, 9/10 L2-3 and 4/6 L1-2med grafts), despite having been grafted lateral to the PS. The fact that most (4/5) L1A grafts contained only unilateral contribution further argues that this bilateral contribution is not a product of accidental grafting of pre-existing PSM cells at the midline, and instead that the rostral limit of the CLE does not encounter the PS midline. A similar argument pertains for lateral CLE grafts (L1-2lat), where 2 out of 3 grafts show exclusively unilateral contribution. Since the mesoderm seen in those grafts is most likely to have been carried along with the graft, they should give an indication of the extent of contamination. The boxplot below shows that this mesoderm constitutes less than 20% of the rostrocaudal axis produced after grafting (estimated by the number of sections colonised posterior to the start of the forelimb bud).

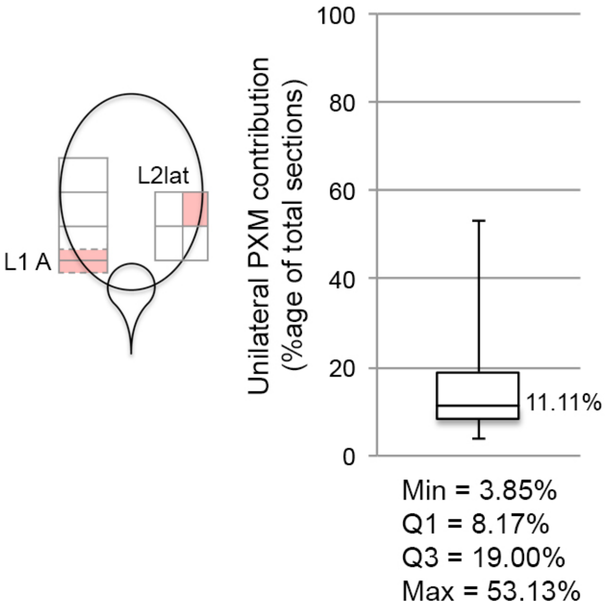

Figure 2. Extent of unilateral paraxial mesoderm contribution in L1A and L2lat grafts. Boxplot shows the distribution of unilateral PXM contribution in L1A and L2lat grafts (*embryos 1.02, 1.03, 1.04, 1.08, 1.09, 2.06, 2.07* and *2.08*, n=8). Unilateral PXM constitutes less than 20% of the rostrocaudal axis produced after grafting (estimated by the number of sections colonised posterior to the start of the forelimb bud). *Embryo 1.02* was the main outlier with 17 sections containing unilateral PXM, however, six of those only contained very few cells contributing to the PXM.

References

1. Cambray N, Wilson V. Two distinct sources for a population of maturing axial progenitors. Development. 2007;134(15):2829-40. Epub 2007/07/06. doi: dev.02877 [pii] 10.1242/dev.02877. PubMed PMID: 17611225.

2. Tam PP. The allocation of cells in the presomitic mesoderm during somite segmentation in the mouse embryo. Development. 1988;103(2):379-90. PubMed PMID: 3224560.

3. Nicolas JF, Mathis L, Bonnerot C, Saurin W. Evidence in the mouse for self-renewing stem cells in the formation of a segmented longitudinal structure, the myotome. Development. 1996;122(9):2933-46. PubMed PMID: 8787766.

4. Tam PP. A study of the pattern of prospective somites in the presomitic mesoderm of mouse embryos. J Embryol Exp Morphol. 1986;92:269-85. Epub 1986/03/01. PubMed PMID: 3723065.

5. Eloy-Trinquet S, Nicolas JF. Cell coherence during production of the presomitic mesoderm and somitogenesis in the mouse embryo. Development. 2002;129(15):3609-19. PubMed PMID: 12117811.
